# Online authentic self-presentation, relatedness need satisfaction, and depression among university students: the moderating role of perceived value of team sports participation in a survey of universities in Shanghai

**DOI:** 10.3389/fpsyg.2026.1864726

**Published:** 2026-08-05

**Authors:** Zhe Zheng, Jiatian Qi, Yue Xi, Yueyi Jia, Qiaoli Xiao, Jingjing Li, Hui Qiu, Bing Liu

**Affiliations:** 1 Physical Education College, Shanghai University, Shanghai, China; 2 School of Economics, Shanghai University, Shanghai, China; 3 School of Physical Education, Shanghai University of Traditional Chinese Medicine, Shanghai, China

**Keywords:** depression, online authentic self-presentation, perceived value of team sports participation, relatedness need satisfaction, university students

## Abstract

**Background:**

With the increasing prevalence of online social interaction, university students often experience insufficient satisfaction of their relatedness needs. Online authentic self-presentation has become a common mode of online communication among university students, and when it becomes excessive or compulsive, it may increase the risk of depression. From the perspective of perceived value of team sports participation, this study examined the mechanism through which online authentic self-presentation influences depression among university students, with a particular focus on the mediating role of relatedness needs and the moderating role of perceived value of team sports participation.

**Methods:**

A questionnaire survey was conducted among 404 university students. Data were collected using the Online Authentic Self-Presentation Scale, the Relatedness Needs Scale, the Perceived Value of Team Sports Participation Scale, and the Depression Scale. Mediation and moderated mediation analyses were performed using the PROCESS macro in SPSS.

**Results:**

Online authentic self-presentation did not significantly predict depression directly, but it exerted a significant indirect effect on depression through relatedness need satisfaction. Relatedness need satisfaction played a significant mediating role in the relationship between online authentic self-presentation and depression. Perceived value of team sports participation significantly moderated both the path from online authentic self-presentation to relatedness need satisfaction and the path from relatedness need satisfaction to depression. Specifically, higher levels of perceived value of team sports participation weakened the positive predictive effect of online authentic self-presentation on relatedness need satisfaction, while strengthening the negative predictive effect of relatedness need satisfaction on depression.

**Conclusion:**

Online authentic self-presentation primarily influences depression among university students through relatedness need satisfaction, and perceived value of team sports participation, as an important protective factor, reshapes this process. Universities should pay greater attention to changes in students’ psychological needs in digital contexts and fully leverage the positive role of perceived value of team sports participation in enhancing relatedness, promoting psychological adjustment, and preventing depression.

## 1 Introduction

Depression among university students has become a global public health challenge. According to a recent systematic review, approximately 25% of university students worldwide experience depressive symptoms to varying degrees (Li et al., 2022). The White Paper on the Mental Health of Chinese University Students (2025) reported that the detection rate of depression among Chinese university students reached as high as 34.7% (Lin et al., 2025). The high prevalence and apparent upward trend of depression in this population have drawn increasing attention from Chinese scholars. Some studies have suggested that the developmental trajectory of Chinese university students is characterized by a relatively typical family-society dual-structure pattern, in which the family and society constitute two relatively independent spheres of socialization (Jin et al., 2026), while the transitional phase from family to society remains comparatively underdeveloped. Although the university campus is itself an important component of the broader social system, Chinese academia generally regards the university stage as a transitional adjustment period between the family and society. This period is not only a critical stage in which students gradually detach themselves from family dependence and move toward independent living, but also a key phase for receiving socialization-oriented education and developing socio-psychological qualities (LaFreniere, 2024). Providing targeted guidance during this stage may not only enhance students’ future social adaptability but also lay an essential foundation for the development of sound personality and healthy socio-psychological functioning. At the same time, some scholars have argued that, after leaving the protection of the family, university students are more likely to experience negative emotions such as anxiety when confronted with academic pressure, social competition, and role transitions, and may cope through maladaptive behaviors such as avoidance and impulsivity (Wong and Shek, 2025). Such adjustment imbalances and behavioral deviations often constitute important antecedents of psychological problems among university students.

Over time, scholars have examined the causes and mechanisms of depression among university students from multiple perspectives, including external factors such as employment pressure (Gao et al., 2020), academic stress, and interpersonal relationships (Liu et al., 2023), as well as internal mechanisms such as psychological resilience and self-identity (Hu and Chen, 2025). With the rapid development of internet technology, online social interaction has gradually become a new means through which university students build interpersonal relationships and has begun to attract growing scholarly attention (Yan et al., 2024). As university years represent a transitional stage toward broader social participation, accompanied by mounting academic demands and social competition, students often experience relatively high levels of loneliness, while an increasingly intense and competitive educational climate makes it difficult for them to find appropriate confidants in real life (Lee et al., 2019). Consequently, online social interaction has emerged as a contemporary pathway through which university students seek psychological compensation and relief from psychological distress (Shen and Wang, 2019).

In recent years, the relationship between university students’ online behaviors and mental health has received increasing scholarly attention. In particular, the continuing rise in depression among university students has prompted educators to reflect on whether the development of prosocial behavior is being compressed and weakened to some extent. Social compensation theory suggests that individuals with relatively limited offline social resources, such as those experiencing stronger loneliness, higher levels of social anxiety, or insufficient real-life social support, are more likely to use the internet as a compensatory tool for deficiencies in offline interpersonal relationships (Chen et al., 2022). Because online interaction offers greater control and less immediate evaluation pressure, individuals with unmet belongingness needs may find it easier to express themselves, feel understood and accepted, and partially compensate for insufficient offline social connection. Online authentic self-presentation, as a typical form of behavior in online social interaction, is a concrete manifestation of this compensatory process. Some studies have shown that online authentic self-presentation is positively associated with self-esteem, life satisfaction, and subjective wellbeing (Bailey et al., 2020; Niu et al., 2015). Other studies have also suggested that online authentic self-presentation may alleviate depression among university students (Wang et al., 2017). However, existing research indicates that the influence of online authentic self-presentation on positive emotional experiences is complex and ambivalent, representing a typical “double-edged sword” phenomenon (Bailey et al., 2020; Jang et al., 2018). On the one hand, online authentic self-presentation may help university students obtain immediate positive emotional experiences and thereby play a role in alleviating depressive feelings. On the other hand, such effects are often limited to short-term emotional compensation. When online social behaviors develop into habitual and sustained patterns, or even excessive dependence, their potential negative implications for university students’ mental health may become increasingly evident. Under such circumstances, individuals may not only fail to obtain stable and enduring positive emotional experiences, but may also weaken real-world interpersonal interaction due to long-term immersion in online sociality, thereby developing a sense of social alienation (Ye et al., 2023). Further, it was found that excessive immersion in online social interaction may impair university students’ ability to recognize emotions in face-to-face communication, thus creating a negative cycle characterized by strengthened virtual connection and weakened real-world connection (Kraut et al., 2002). Taken together, the effect of online authentic self-presentation on depression among university students remains controversial, and its underlying mechanism warrants further investigation.

According to need to belong theory, the need to belong is a fundamental psychological need through which individuals seek interpersonal connection and group acceptance, and the extent to which this need is satisfied is directly associated with the risk of depression (Baumeister and Leary, 1995). As research on depression has continued to deepen, the focus has gradually shifted from physiological and neurobiological mechanisms in university students to the social-acceptance and prosocial-behavior mechanisms underlying depressive symptoms. For example, educational psychology studies have encouraged students to engage in collaborative projects to enhance cooperative competence, as well as to participate in innovation and entrepreneurship competitions in order to strengthen competitiveness through collective intelligence and teamwork (Meng, 2025; Peng et al., 2025). These studies provide contemporary scholars with a broader basis for further examining the role and mechanisms of collective behavioral engagement in shaping university students’ sense of social acceptance and group inclusion.

Participation in team sports requires teamwork and may strengthen a sense of group belonging. At the same time, the depression-buffering effects associated with physical activity may help reduce depressive symptoms by supplementing the satisfaction of relatedness needs. By providing empirical evidence on how university students may cultivate a stronger sense of social belonging, this study ultimately seeks to inform educational efforts aimed at promoting healthier lifestyles among this population.

## 2 Literature review

### 2.1 Online authentic self-presentation and depression

Previous studies have shown that certain forms of active and socially oriented social networking site use may be associated with greater social capital, perceived social support, social adjustment, and psychological wellbeing among university students (Gray et al., 2013; Zhang et al., 2023). Authentically presenting self-related information and engaging in online interactions through emotional expression and self-disclosure may facilitate self-expression, promote social adjustment, and support the development of interpersonal relationships, thereby contributing positively to the maintenance of mental health (Scott et al., 2022). From this perspective, online authentic self-presentation is not necessarily a negative behavior. Rather, it may provide university students with opportunities for self-expression, relational validation, and social connectedness.

However, the potential psychological implications of online authentic self-presentation should also be considered in light of the social displacement hypothesis. This hypothesis suggests that online social interaction may occupy time and psychological resources that would otherwise be devoted to offline interpersonal communication, thereby weakening real-life social connectedness (Valkenburg and Peter, 2007). For university students navigating a critical period of identity development, reliance on online self-presentation and communication for emotional expression or interpersonal validation may, under conditions such as social anxiety or loneliness, be associated with lower face-to-face self-disclosure, reduced engagement in offline social activities, or weaker perceived social connectedness (Nowland et al., 2017; Weidman et al., 2012). In this sense, online authentic self-presentation may not always be linked to positive psychological adjustment. Its association with depressive symptoms may depend on whether online expression complements or competes with offline social relationships.

The differential susceptibility to media effects model further suggests that the relationship between media use and individuals’ psychological outcomes is not homogeneous (Valkenburg and Peter, 2013), but is jointly shaped by individual characteristics, developmental stage, and social environment. In other words, the same form of online authentic self-presentation may have different psychological meanings for different individuals. For some individuals, it may be associated with positive self-expression and relationship maintenance; for others, it may be linked to greater social evaluation pressure, relational uncertainty, or negative emotional experiences. This may be particularly relevant for university students, who are still in a critical stage of self-identity formation and interpersonal development. Their authentic expression online may therefore be more susceptible to external feedback and social comparison. Uses and gratifications theory (Tran and Diep, 2025; Whiting and Williams, 2013) also provides a complementary explanation for this relationship. This theory suggests that individuals do not passively receive media effects; rather, they use social media to satisfy different psychological and social needs, such as relationship maintenance, emotional expression, recognition seeking, entertainment, and information acquisition. Thus, even when individuals engage in the same behavior of online authentic self-presentation, their motivations and psychological outcomes may differ. Some individuals may obtain support and connectedness through authentic expression, whereas others may be more concerned with likes, comments, and social approval. When online authentic self-presentation is more closely tied to external evaluation and the need for relational validation, it may be positively associated with depressive symptoms.

Social support theory posits that social support can serve as a protective resource against depression, but this premise depends on the presence of effective and positive social connections (Sharifian and O'Brien, 2019). When university students engage in online authentic self-presentation for extended periods, the support they obtain may not necessarily be high-quality social support; instead, they may be exposed to multiple forms of negative social feedback (Epifanio and Gallegos, 2025). On the one hand, the unreserved expression of negative emotions and personal difficulties may challenge individuals’ sense of self-worth, elicit feelings such as shame and anxiety, and thereby be associated with higher depressive symptoms (Yang et al., 2023). On the other hand, the weak-tie nature of online social interaction means that feedback is often fragmented and superficial (Ellison et al., 2007). A strong reliance on online authentic self-presentation to obtain emotional support may lead individuals to develop biased perceptions of social support, mistaking online “likes” and “comments” for deep interpersonal communication. This may cause them to overlook face-to-face emotional exchanges in real life, and the lack of genuine social support may ultimately be associated with more severe depressive symptoms (Hunt et al., 2018).

Previous studies have suggested that online authentic self-presentation may alleviate depressive symptoms by facilitating self-expression, social support, and interpersonal connectedness (Wang et al., 2017; Wright et al., 2012). However, limited attention has been paid to university students, a population undergoing a critical stage of identity formation, and few studies have further examined the potential risks associated with self-presentation in this specific group (Gutiérrez Arenas et al., 2024). Taken together, the existing literature shows that the relationship between online authentic self-presentation and depressive symptoms remains inconsistent. Some studies suggest a positive association, whereas others indicate a negative association. However, given that a relatively larger body of literature has reported a positive association, the present study proposes hypothesis 1 (H1): Online authentic self-presentation positively predicts depression among university students.

### 2.2 The mediating role of relatedness need satisfaction

Relatedness needs refer to individuals’ psychological tendency to seek acceptance and recognition from a group, with the core aim of establishing, maintaining, and consolidating interpersonal connections through various means (Baumeister and Leary, 1995). As an important socio-psychological experience, a sense of belonging exerts a significant influence on individuals’ physical and mental health as well as their subjective wellbeing. In the present study, relatedness need satisfaction refers to the extent to which participants perceive their need for belonging and interpersonal connectedness to be fulfilled. In the context of online social interaction, social compensation theory suggests that when individuals experience difficulties in offline interaction or have insufficient access to real-life belonging resources, they are more likely to turn to online spaces to compensate for their needs for social connection and belonging (Zinchenko et al., 2022). The anonymity, lower perceived risk, and controllability of online communication may reduce barriers to interaction, making individuals more willing to engage in authentic expression and seek relational validation (Hollenbaugh and Everett, 2013). Meanwhile, the online psychological need satisfaction advantage perspective emphasizes that the accessibility and convenience of online social interaction can supplement belonging needs that remain insufficiently fulfilled in real life. Through immediate feedback and sustained interaction, online social interaction may generate positive emotional experiences and alleviate social anxiety and interpersonal distress (Liu et al., 2020).

Based on the foregoing arguments, appropriate online authentic self-presentation can be understood as an important way for university students to actively seek belonging and relational connection on social media. To a certain extent, authentic self-presentation helps enhance self-consistency and interactional credibility, promotes others’ understanding, responsiveness, and acceptance, and thereby strengthens individuals’ sense of recognition and relational security, encouraging them to invest more effort in maintaining interpersonal relationships (Li et al., 2021). When individuals perceive acceptance and support in online interactions, such support may strengthen their self-esteem and alleviate feelings of loneliness, thereby potentially buffering against depressive symptoms (Lin et al., 2020; Zhang et al., 2022). Conversely, when relatedness needs remain unmet over time or relational affirmation is insufficient, individuals are more likely to experience feelings of exclusion and emotional distress, thereby increasing the risk of depression (Dienst et al., 2023). Accordingly, it can be inferred that online authentic self-presentation does not directly determine the level of depression, but is more likely to influence depression indirectly by shaping individuals’ experiences of belonging and the state of their relational connectedness. Based on this reasoning, this study proposes Hypothesis 2 (H2): Relatedness need satisfaction mediate the relationship between online authentic self-presentation and depression among university students.

### 2.3 The moderating role of perceived value of team sports participation

University students commonly experience loneliness and insufficient offline prosocial behavior, which makes them more likely to rely on online authentic self-presentation to obtain emotional support and a sense of belonging (Hood et al., 2017; Morahan-Martin and Schumacher, 2003). However, excessive dependence on online interaction may intensify social alienation in real life and fail to fundamentally reduce the risk of depression (Primack et al., 2017). Compensatory internet use theory suggests that collective activities conducive to physical and mental wellbeing can effectively help individuals shift from online engagement back to real-world social life, thereby reducing the likelihood of loneliness and depression (Cipolletta et al., 2024). Some scholars further argue that team sports, as a form of collective activity oriented toward unity, cooperation, and the cultivation of willpower, can serve as an effective means of compensatory internet use, thereby facilitating individuals’ return to their authentic selves in real-life contexts (Wei et al., 2025). A salient feature of team sports participation is its emphasis on collective strength and teamwork. Through collaboration, individuals and groups are able to build positive cooperative relationships and mutual understanding, while also fostering students’ cooperative spirit, team consciousness, and interpersonal qualities such as understanding, tolerance, and trust (Liu et al., 2025). Social support theory posits that team sports participation, as an important source of social support, creates authentic social settings in which university students can receive immediate and tangible emotional support; accordingly, it has long been regarded as an effective pathway for alleviating depression (Zhou and Lin, 2025). Research has shown that participation in physical activity, especially team sports, can strengthen individuals’ sense of collective honor and significantly enhance their group identity through team interaction (Ni et al., 2025). Compared with the feedback obtained through online self-presentation, such support in real-world interaction carries greater emotional salience and can more directly strengthen university students’ sense of connectedness in actual social life (Su et al., 2025). Some scholars have also noted that team sports often involve stable teammates and sustained companionship over time; because of the relative stability of the team, such companionship can provide continuous and dependable emotional bonds (Wan et al., 2025). In addition, the experiences of cooperation and success in team sports can directly enhance university students’ sense of self-worth (Chen et al., 2026), further strengthening the satisfaction of their relatedness needs in real life and reducing the likelihood that they will rely excessively on online authentic self-presentation to compensate for unmet belonging needs. In this way, team sports participation may help university students re-engage with real-life contexts and promote their prosocial behavior (Seidman, 2013). Therefore, perceived value of team sports participation may play a moderating role in the relationship between online authentic self-presentation and relatedness need satisfaction.

According to need to belong theory (Hagerty et al., 1992), relatedness need reflects individuals’ psychological wellbeing through the quality of their interpersonal relationships. Positive external factors may further enhance this process by strengthening the association between relatedness need and psychological adjustment (Baumeister and Tice, 1990). For university students with higher levels of participation in team sports, the interpersonal trust and emotional support accumulated within the team may function as a psychological buffer (Chen et al., 2026; Shi et al., 2026b). Moreover, individuals who regularly engage in physical activity are more likely to develop close relationships with others (Liu et al., 2026). This is particularly evident in team sports, where the horizontal interpersonal interactions among participants are especially conducive to promoting social communication and establishing as well as maintaining stable interpersonal relationships (He et al., 2026). Even when relatedness needs are not fully satisfied, other real-world resources may still help alleviate negative emotions. A substantial body of research has also shown that participation in team sports can effectively reduce stress responses and enhance social engagement, thereby lowering the risk of depression (Cooney et al., 2013; Panza et al., 2020). Therefore, perceived value of team sports participation may play a moderating role in the relationship between relatedness need satisfaction and depression.

**Figure 1:**
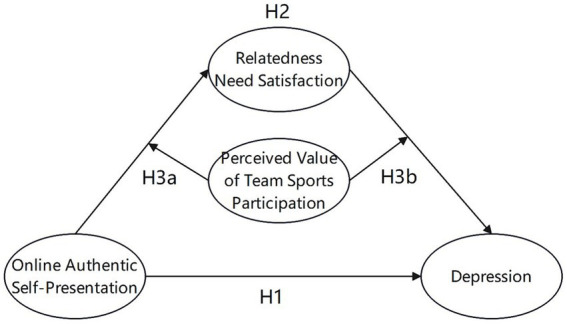
Hypothesized model of the pathways through which online authentic self-presentation influences depression. *H1* = direct effect; *H2* = mediating effect; *H3a* and *H3b* = moderating effects.

Based on the above literature, this study proposes Hypothesis 3 (H3): Perceived value of team sports participation moderates the pathway linking online authentic self-presentation, relatedness need satisfaction, and depression. Specifically, H3a states that perceived value of team sports participation moderates the relationship between online authentic self-presentation and relatedness need satisfaction; H3b states that perceived value of team sports participation moderates the relationship between relatedness need satisfaction and depression. The overall hypothetical model of the present study is shown in Figure 1.

## 3 Methods

### 3.1 Subjects and procedures

Shanghai, as a leading center of socioeconomic development, higher education, and technological innovation in China, is also one of the most highly informatized cities in the country. Although it offers university students abundant opportunities for development, students in Shanghai also face substantially greater employment and competitive pressures than those in many other cities (Sun, 2024). As a result, a considerable proportion of university students, constrained by the pace of academic and daily life, find it difficult to identify appropriate confidants in real life. This, in turn, contributes to more frequent engagement in online social interaction and stronger feelings of loneliness among students in Shanghai universities, thereby further increasing the risk of depression. Therefore, the characteristics of this sample are not only conducive to revealing the mechanisms linking online authentic self-presentation and depression, but also provide empirical insights into how online social interaction affects university students’ mental health in the context of China’s rapid social development. Participants were recruited using a convenience sampling approach from universities in Shanghai. Questionnaires were distributed through the student affairs offices of universities located in the Yangpu University Town area of Shanghai, including Fudan University, Tongji University, Shanghai University of Finance and Economics, Shanghai International Studies University, the University of Shanghai for Science and Technology, and Shanghai University of Sport. Data were collected through the online survey platform Wenjuanxing. After reading and signing the informed consent form, participants proceeded to complete the formal questionnaire. To reduce response tendency and potential common method bias, the questionnaire was completed anonymously, participants were informed that there were no right or wrong answers, standardized instructions were provided, and reverse-worded items were included where appropriate. In this study, a total of 461 participants completed the questionnaire survey. In accordance with the questionnaire quality-control criteria (Curran, 2016), and with reference to the distribution of response times obtained in the pilot survey conducted among university students, questionnaires completed in less than 110 s were classified as invalid and excluded, resulting in the removal of 31 cases. Questionnaires were further screened for response quality before data analysis. Responses were excluded if they showed clear inconsistencies in reverse-coded items or obvious patterned responding across randomly arranged items (Desimone et al., 2015; Meade and Craig, 2012). Because several reverse-coded items were included and some items were randomly arranged, identical responses to five consecutive items were considered together with reverse-item consistency and overall response patterns, rather than as a single automatic exclusion criterion. This procedure led to the exclusion of a further 26 invalid cases. A total of 404 valid questionnaires were ultimately retained, yielding a valid response rate of 87.6%. The final sample consisted of 206 male students and 198 female students, indicating a broadly balanced gender distribution. The study protocol was approved by the Ethics Committee of Shanghai University (ECSHU 2025–019).

### 3.2 Measurement tools

#### 3.2.1 Online authentic self-presentation scale

The present study adopted the Positive Self-Presentation Scale and the Authentic Self-Presentation Scale originally developed by Kim and Lee (2011) for Facebook-based research, using the Chinese version of the Social Networking Site Self-Presentation Questionnaire as revised by Niu et al. (2015) for the Chinese context. This version has demonstrated satisfactory psychometric properties and has been applied in multiple studies involving Chinese university students (Tian et al., 2022; Wang et al., 2026). The full questionnaire contains 20 items and comprises two dimensions: positive self-presentation (16 items) and authentic self-presentation (4 items). Consistent with the purpose of the present study, only the authentic self-presentation dimension was used. Example items include: “I post pictures on social networking sites that show my true self (e.g., real photos of myself participating in activities)” and “On social networking sites, I tend to express my real thoughts and feelings.” All items were rated on a five-point Likert scale ranging from 1 (“strongly disagree”) to 5 (“strongly agree”). In this study, the scale showed satisfactory reliability and convergent validity, with a Cronbach’s *α* of 0.845, a composite reliability of 0.875, and an average variance extracted (AVE) of 0.636.

#### 3.2.2 Relatedness needs scale

The present study used the Basic Psychological Needs Scale originally developed by Gagné (2003). The scale has been widely used in studies involving Chinese university students (Chen et al., 2014). The full scale consists of 19 items and includes three dimensions: autonomy need, relatedness need, and competence need. In line with the purpose of the present study, only the relatedness need dimension was selected for measurement. Example items include: “I am very friendly toward people who are willing to interact with me” and “In daily life, people who interact with me often take my feelings into consideration.” All items were rated on a five-point Likert scale ranging from 1 (“strongly disagree”) to 5 (“strongly agree”). In this study, the scale showed satisfactory reliability and convergent validity, with a Cronbach’s α of 0.868, a composite reliability of 0.878, and an AVE of 0.546.

#### 3.2.3 Perceived value of team sports participation scale

Perceived value of team sports participation refers to students’ subjective evaluations of the value, benefits, responsibility, identification, and integration associated with participating in team sports. In this study, team sports participation was measured using the questionnaire developed by Shi et al. (2026) for the context of team sports participation among Chinese university students. The scale consists of nine items, including statements such as “Team sports participation helps cultivate members’ sense of social identity” and “Team sports participation helps strengthen individuals’ sense of responsibility.” All items were rated on a five-point Likert scale ranging from 1 (“strongly disagree”) to 5 (“strongly agree”). In the present study, the scale demonstrated good internal consistency reliability and convergent validity, with a Cronbach’s *α* of 0.949, a composite reliability of 0.942, and an AVE of 0.642.

#### 3.2.4 Depression scale

The present study employed the Center for Epidemiologic Studies Depression Scale originally developed by Radloff (1977). The Chinese version adapted and examined by Chen et al. (2009) was used in the present study. The CES-D has demonstrated satisfactory psychometric properties among Chinese university students and has been applied in both cross-sectional and longitudinal studies of this population (Jiang et al., 2019; Song et al., 2020). Although the original CES-D assesses depressive symptoms experienced during the past week, previous research has shown that extending the reference period to the past month produces comparable screening performance. Therefore, the present study used a one-month timeframe to assess participants’ recent depressive symptoms (Chiu et al., 2010). The scale consists of 10 items and covers four dimensions: depressed affect, positive affect, somatic symptoms and retarded activity, and interpersonal problems. Example items include: “In the past month, I often felt hopeless about the future” and “In the past month, I often felt lonely.” All items were rated on a five-point Likert scale ranging from 1 (“strongly disagree”) to 5 (“strongly agree”). In the present study, the scale demonstrated good internal consistency reliability and convergent validity, with a Cronbach’s *α* of 0.935, a composite reliability of 0.938, and an AVE of 0.602.

### 3.3 Data processing

All statistical analyses were conducted using SPSS Statistics 26.0 and the PROCESS macro version 4.2. Descriptive statistics were first calculated for the main study variables, and Pearson correlation analyses were used to examine the bivariate associations among online authentic self-presentation, relatedness need satisfaction, depressive symptoms, and perceived value of team sports participation. Preliminary analyses showed no significant between-group differences in the main demographic characteristics of the sample (*p* > 0.05), suggesting that the groups were broadly comparable.

The moderated mediation model was tested using Model 58 of the PROCESS macro. Online authentic self-presentation was entered as the independent variable, relatedness need satisfaction as the mediator, depressive symptoms as the dependent variable, and perceived value of team sports participation as the moderator. A bias-corrected bootstrapping procedure with 5,000 resamples was used to estimate the indirect and conditional indirect effects. Effects were considered statistically significant when the 95% confidence interval did not include zero. Before creating the interaction terms, all continuous variables involved in the interaction analyses were mean-centered to reduce multicollinearity and facilitate interpretation.

A sensitivity power analysis was added to justify the adequacy of the sample size. Using a linear multiple regression model with *R*^2^ increase, with *α* = 0.05, statistical power = 0.80, one tested predictor, eight total predictors in the most complex regression equation, and the final valid sample size of *N* = 404, the minimum detectable effect size was *f*^2^ = 0.0195, corresponding to an incremental explained variance of approximately Δ*R*^2^ = 0.0191. This indicates that the current sample size was adequate to detect small interaction effects.

## 4 Results

### 4.1 Common method deviation analysis

To reduce the potential influence of common method bias, several procedural controls were adopted during questionnaire administration. Specifically, participants completed the questionnaire anonymously, were informed that there were no right or wrong answers, received standardized instructions, and some items were randomly arranged. Harman’s single-factor test was used as a preliminary diagnostic check for common method bias. The results showed that four factors had eigenvalues greater than 1, and the first factor accounted for 35.8% of the total variance, which was below the commonly used threshold of 40%. This result suggests that common method bias was not statistically evident in the present study, although it cannot be fully ruled out.

### 4.2 Descriptive statistics

In terms of academic standing, the participants were relatively evenly distributed across the four undergraduate years. Specifically, 92 students were freshmen (22.8%), 113 were sophomores (27.9%), 108 were juniors (26.7%), and 91 were seniors (22.6%). Regarding age distribution, 173 were aged 18–20 years (42.8%), 155 were aged 20–22 years (38.4%), and 76 were older than 22 years (18.8%) (Table 1).

**Table 1:** Demographic characteristics (*N* = 404).

Variable	Classification	Sample	Proportion (%)
Gender	Male	206	50.9
	Female	198	49.1
Grade	Freshman	92	22.8
	Sophomore	113	27.9
	Junior	108	26.7
	Senior	91	22.6
Age	18 ≤ Age < 20	173	42.8
	20 ≤ Age < 22	155	38.4
	Age ≥ 22	76	18.8

### 4.3 Correlation analysis and square root of the AVE

The results of the correlation analysis (Table 2) showed that online authentic self-presentation was not significantly correlated with depression among university students (*r* = −0.08, *p* > 0.05), but was significantly and positively correlated with relatedness need satisfaction (*r* = 0.311, *p* < 0.001). Relatedness need satisfaction, in turn, were significantly and negatively correlated with depression among university students (*r* = −0.283, *p* < 0.001). In addition, perceived value of team sports participation was significantly and negatively correlated with online authentic self-presentation (*r* = −0.352, *p* < 0.001), significantly and positively correlated with relatedness need satisfaction (*r* = 0.449, *p* < 0.001), and significantly and negatively correlated with depression among university students (*r* = −0.381, *p* < 0.001). Further tests of discriminant validity indicated that the square root of the AVE for each variable was greater than its correlations with the other variables, suggesting that the constructs examined in the present study demonstrated good discriminant validity.

**Table 2:** Correlation analysis and the square root of AVE (*N* = 404).

Variable	M	SD	Online authentic self-presentation	Relatedness need satisfaction	Depression	Perceived value of team sports participation
Online authentic self-presentation	3.46	0.91	**0.797** ^a^			
Relatedness need satisfaction	3.40	0.83	0.311^***^^b^	**0.739**		
Depression	2.68	0.88	−0.080	−0.283^***^	**0.776**	
Perceived value of team sports participation	2.96	1.04	−0.352^***^	0.449^***^	−0.381^***^	**0.801**

*N* = 404. M, mean; SD, standard deviation; AVE, average variance extracted.

a Bold values on the diagonal represent the square roots of AVE, and values below the diagonal represent Pearson’s correlation coefficients.

b ***p < 0.001.

### 4.4 Testing the mediating role of relatedness need satisfaction

The present study used Model 4 of the PROCESS macro for SPSS to test the mediating effect, and further examined the mediating role of relatedness need satisfaction in the relationship between online authentic self-presentation and depression using the Bootstrap method proposed by Hayes (2012). The results (Table 3) showed that, after controlling for gender, grade, and age, the 95% confidence interval (CI) for the direct effect of online authentic self-presentation on depression included zero, indicating that its direct predictive effect on depression was not statistically significant. By contrast, the 95% CI for the indirect effect of online authentic self-presentation on depression through relatedness need satisfaction did not include zero, suggesting that relatedness need satisfaction played a significant mediating role in this relationship. These findings indicate that online authentic self-presentation does not directly predict depression among university students, but instead primarily influences depression through the mediating role of relatedness need satisfaction. Therefore, Hypothesis 1 (H1) was not supported, whereas Hypothesis 2 (H2) was supported.

**Table 3:** Total, direct, and indirect effects of online authentic self-presentation on depression via relatedness need satisfaction (*N* = 404).

Variable	Effect size	SE	LLCI	ULCI	Effect proportion
Total effect	−0.164	0.048	−0.259	−0.069	
Direct effect	−0.078	0.049	−0.174	0.018	
Indirect effect	−0.086	0.020	−0.129	−0.050	52.44%

### 4.5 Testing the moderating role of perceived value of team sports participation

The moderating effect was examined using Model 58 of the PROCESS macro in SPSS. As shown in Table 4, the results provided overall empirical support for the proposed moderated mediation model. In the regression model with relatedness need satisfaction as the dependent variable, online authentic self-presentation significantly and positively predicted relatedness needs (*B* = 0.11, SE = 0.05, *t* = 2.36, *p* < 0.05), and perceived value of team sports participation also had a significant positive predictive effect on relatedness need satisfaction (*B* = 0.33, SE = 0.04, *t* = 8.94, *p* < 0.001). In addition, the interaction term between online authentic self-presentation and perceived value of team sports participation significantly predicted relatedness needs (*B* = −0.12, SE = 0.04, *t* = −2.97, *p* < 0.01), indicating that perceived value of team sports participation moderated the path from online authentic self-presentation to relatedness need satisfaction. Specifically, as the level of perceived value of team sports participation increased, the positive predictive effect of online authentic self-presentation on relatedness need satisfaction became weaker.

**Table 4:** Tests of the moderated mediation model (*N* = 404).

Variable	Need to belong satisfaction			Depression		
	*B*	SE	*t*	*B*	SE	*t*
Constant	0.13	0.22	0.60	2.81	0.25	11.27***
Gender	0.05	0.07	0.73	−0.12	0.08	−1.46
Grade	0.16	0.06	2.41*	−0.03	0.07	−0.37
Age	−0.17	0.09	−1.93	0.04	0.10	0.45
Level of team sports participation	−0.01	0.02	−0.52	0.01	0.02	0.39
Online authentic self-presentation	0.11	0.05	2.36*	−0.03	0.05	−0.68
Perceived value of team sports participation	0.33	0.04	8.94***	−0.23	0.05	−4.94***
Relatedness need satisfaction				−0.22	0.06	−3.90***
Online authentic self-presentation × perceived value of team sports participation	−0.12	0.04	−2.97***			
Relatedness need satisfaction × perceived value of team sports participation				−0.17	0.05	−3.45**
*F*	20.377***			12.117***		
*R* ^2^	0.265			0.197		

*N* = 404. *B*, unstandardized regression coefficient; SE, standard error. Gender, grade, age, and level of team sports participation were included as control variables. Interaction terms were calculated using mean-centered variables. *R*^2^ represents the proportion of explained variance.

***p < 0.001, **p < 0.01, *p < 0.05.

In the regression model with depression as the dependent variable, the direct predictive effect of online authentic self-presentation on depression was not significant (*B* = −0.03, SE = 0.05, *t* = −0.68, *p* > 0.05). By contrast, both perceived value of team sports participation (*B* = −0.23, SE = 0.05, *t* = −4.94, *p* < 0.001) and relatedness need satisfaction (*B* = −0.22, SE = 0.06, *t* = −3.90, *p* < 0.001) significantly and negatively predicted depression. Moreover, the interaction term between relatedness needs and perceived value of team sports participation significantly predicted depression (*B* = −0.17, SE = 0.05, *t* = −3.45, *p* < 0.01), indicating that perceived value of team sports participation also moderated the path from relatedness need satisfaction to depression. More specifically, the higher the level of perceived value of team sports participation, the stronger the negative predictive effect of relatedness need satisfaction on depression. In other words, a high level of perceived value of team sports participation further strengthened the protective effect of relatedness need satisfaction against depression.

**Figure 2:**
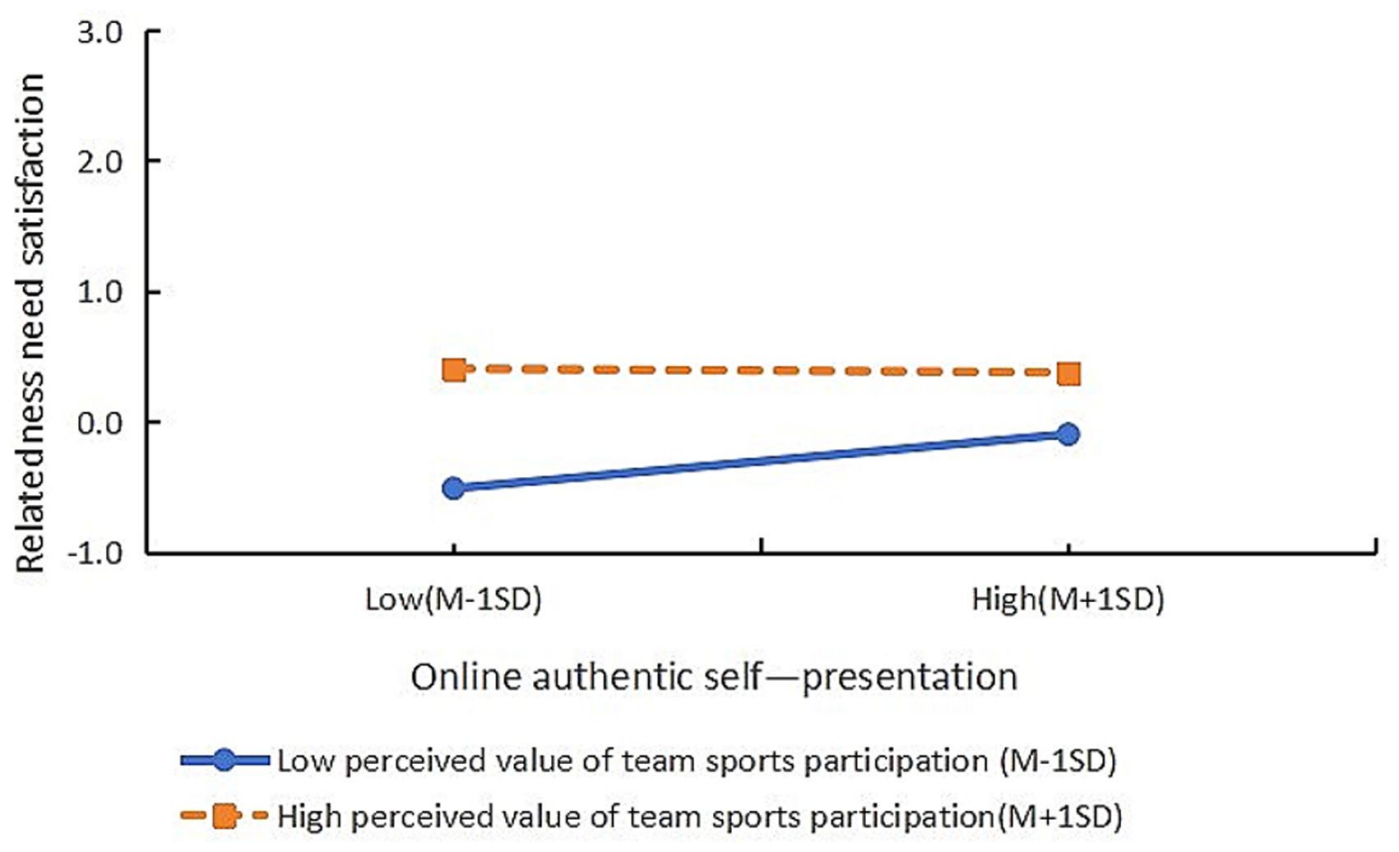
Moderating effect of perceived value of team sports participation on the association between online authentic self-presentation and relatedness need satisfaction.

To further examine the moderating effect of perceived value of team sports participation on the relationship between online authentic self-presentation and relatedness need satisfaction, a simple slope analysis was conducted. Perceived Value of team sports participation was divided into low-level (M − 1SD) and high-level (M + 1SD) groups based on one standard deviation below and above the mean. The results (Figure 2; Table 5) showed that under the condition of low perceived value of team sports participation, online authentic self-presentation significantly and positively predicted relatedness need satisfaction; however, under the condition of high perceived value of team sports participation, this positive predictive effect was attenuated. These findings indicate that perceived value of team sports participation significantly and negatively moderates the relationship between online authentic self-presentation and relatedness need satisfaction.

**Table 5:** Moderating effect of perceived value of team sports participation on the relationship between online authentic self-presentation and relatedness need satisfaction.

Perceived value of team sports participation	Effect	SE	*t*	*p*	LLCI	ULCI
Low	0.23	0.05	4.54	0.000	0.13	0.33
High	−0.02	0.07	−0.23	0.819	−0.15	0.12

**Figure 3:**
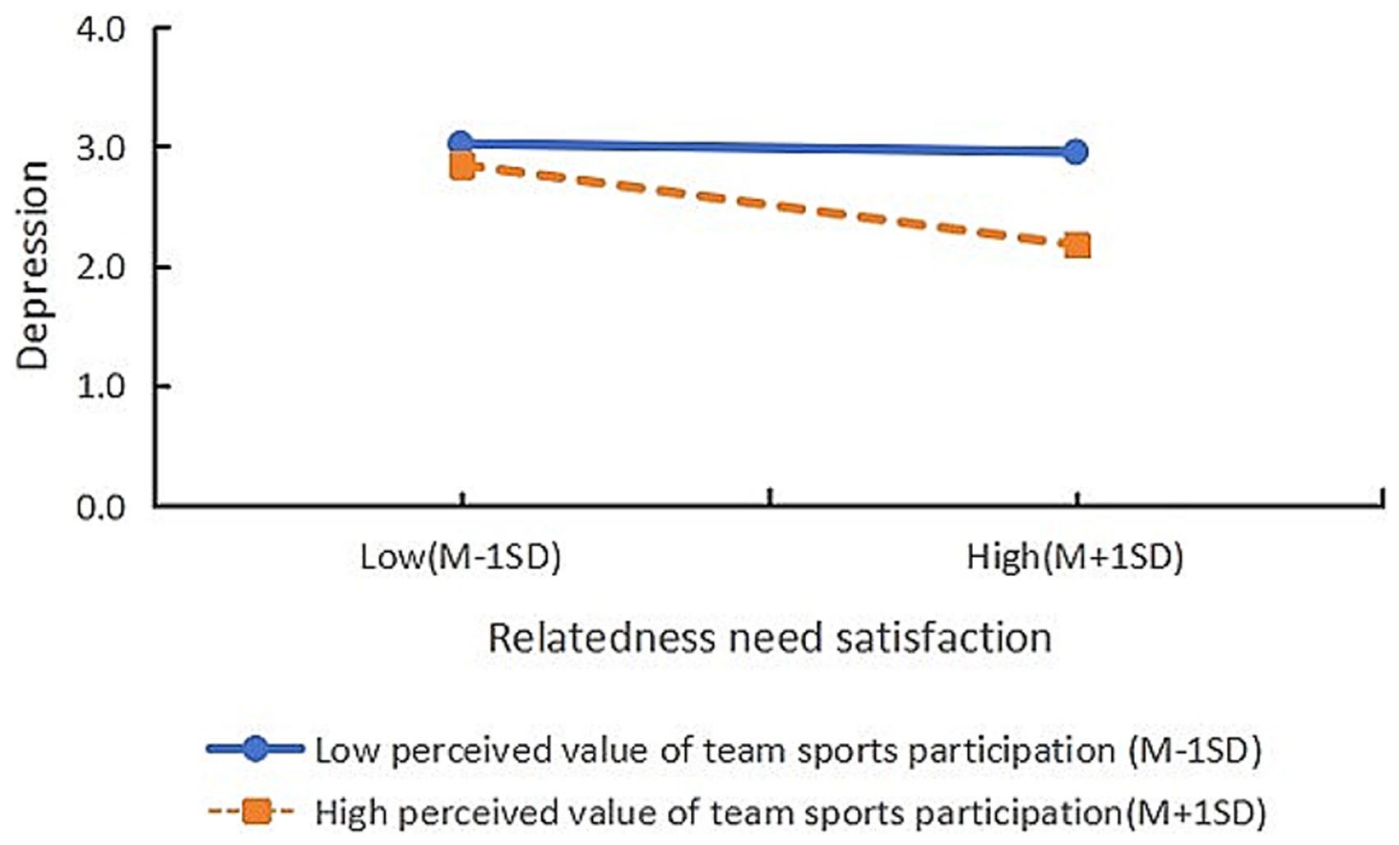
The moderating effect of perceived value of team sports participation on the relationship between relatedness need satisfaction and depression.

To further examine the moderating effect of perceived value of team sports participation on the relationship between relatedness need satisfaction and depression, a simple slope analysis was conducted. The results (Figure 3; Table 6) showed that under the condition of low perceived value of team sports participation, the negative predictive effect of relatedness need satisfaction on depression was relatively weak; however, under the condition of high perceived value of team sports participation, this negative predictive effect was significantly stronger. These findings indicate that perceived value of team sports participation significantly and negatively moderates the relationship between relatedness need satisfaction and depression. In other words, the higher the level of perceived value of team sports participation, the stronger the protective effect of relatedness need satisfaction against depression.

**Table 6:** Moderating effect of perceived value of team sports participation on the relationship between relatedness need satisfaction and depression.

Perceived value of team sports participation	Effect	SE	*t*	*p*	LLCI	ULCI
Low	−0.04	0.07	−0.62	0.537	−0.17	0.09
High	−0.41	0.09	−4.62	0.000	−0.58	−0.23

## 5 Discussion

### 5.1 Online authentic self-presentation and depression

The results showed that, after accounting for the moderating role of the perceived value of team sports participation, the direct association between online authentic self-presentation and depressive symptoms among university students was not statistically significant (*B* = −0.03, *p* > 0.05); therefore, Hypothesis 1 (H1) was not supported. This finding suggests that online authentic self-presentation itself may not necessarily constitute a direct risk factor for depressive symptoms. Rather, the strength and direction of its association with depressive symptoms may be jointly conditioned by individuals’ psychological need satisfaction and specific social contexts (Wong and Hamza, 2025). To some extent, this finding extends the existing understanding of the relationship between online authentic self-presentation and depression. Whether and to what extent online authentic self-presentation affects depression among university students must be examined within a more comprehensive psychological-social mechanism. Therefore, the lack of support for this direct path does not imply that online authentic self-presentation is unrelated to depression; instead, it indicates that its mode of influence may be more complex than previously suggested in the literature.

From the perspective of self-determination theory, online authentic self-presentation is not itself a direct risk variable for depression, but rather a form of self-expression that may influence the satisfaction of individuals’ basic psychological needs. Only when such expression is linked to the satisfaction of relatedness needs and to a stronger sense of interpersonal connectedness and acceptance may its psychological relevance be further reflected in depressive symptoms (Bunker et al., 2024). Therefore, the non-significant direct association between online authentic self-presentation and depressive symptoms in the present study suggests that its role may be indirectly reflected through psychological mechanisms such as relatedness need satisfaction, rather than through a simple linear association with depressive symptoms.

### 5.2 Mediating role of relatedness need satisfaction

The present study systematically elucidated the mechanism through which online authentic self-presentation influences depression among university students by examining the mediating effect of relatedness need satisfaction. The results showed that relatedness need satisfaction played a significant mediating role in this relationship (*B* = −0.22, *p* < 0.05), accounting for 52.44% of the relative effect. This finding not only supports Hypothesis 2 (H2), but also provides new empirical evidence for reconciling inconsistencies in previous research and advancing the study of university students’ mental health in the digital age.

The decomposition of effects further showed that the total effect of online authentic self-presentation on depression among university students was −0.164 [95% CI = (−0.259, −0.069)], indicating a significant overall association between the two variables. After relatedness need satisfaction was included in the model, the direct effect was −0.078 [95% CI = (−0.174, 0.018)], whereas the indirect effect was −0.086 [95% CI = (−0.129, −0.050)]. Notably, the indirect effect accounted for 52.44% of the total effect, exceeding one half, which suggests that the impact of online authentic self-presentation on depression was mainly realized through the satisfaction of relatedness need satisfaction. These results indicate that relatedness need satisfaction is not a peripheral factor, but rather a key mediating variable linking online self-presentation and mental health. This finding also provides an important entry point for understanding the mechanisms underlying depression among university students in digital contexts.

In the first half of the mediating pathway, namely the link between online authentic self-presentation and relatedness need satisfaction, the findings of the present study are broadly consistent with the needs gratification advantages of online communication perspective, while also extending previous understandings of the function of online self-presentation to some extent. Prior research has often treated online self-presentation as a passive form of compensation following frustration in offline social interaction. By contrast, the present findings suggest that online authentic self-presentation may serve as an important means through which university students actively construct a sense of belonging. In offline social contexts, university students often inhibit authentic expression because of concerns about negative evaluation and impression management, which makes it difficult for them to obtain deep relational acceptance. In comparison, the online environment, with its trans-spatiotemporal nature, greater flexibility of expression, and relatively lower pressure of evaluation, provides a safer space for the presentation of the authentic self. In this process, individuals are more likely to receive responses, understanding, and resonance, and to satisfy their relatedness needs through interpersonal connections grounded in shared interests, personal characteristics, and emotional identification (Baumeister and Leary, 1995). Therefore, online authentic self-presentation is not merely a simple compensation for insufficient offline belonging, but may, to some extent, reshape the way university students fulfill their relatedness needs.

In the second half of the mediating pathway, namely the link between relatedness need satisfaction and depression, the findings of the present study further indicate that the satisfaction of relatedness needs constitutes an important psychological mechanism for reducing depression among university students. This finding is consistent with the perspectives of self-determination theory (Deci and Ryan, 2000) and the resilience resource model (Mestre et al., 2017). As one of the basic psychological needs, the extent to which relatedness needs are satisfied directly influences individuals’ sense of self-worth, relational security, and emotional experience. When university students perceive themselves as being accepted by a group, understood by significant others, and embedded in relatively stable interpersonal connections, their feelings of loneliness, helplessness, and social alienation are significantly reduced (Priestley et al., 2025). These negative experiences, in turn, are important psychological antecedents of depression. More specifically, a higher level of relatedness need satisfaction may also foster a positive cycle of interpersonal interaction. On the one hand, a sense of belonging helps enhance individuals’ self-efficacy and reduces their negative appraisals of stressful events. On the other hand, stable interpersonal support facilitates the accumulation of psychological resilience resources, thereby strengthening individuals’ capacity to cope with adverse life events such as academic competition and employment pressure (Duraku et al., 2025). Taken together, the satisfaction of relatedness needs exerts a psychologically protective effect through the dual mechanisms of emotional buffering and resource accumulation, thereby reducing the risk of depression.

The significance of the present study also lies in its provision of a new perspective for explaining the inconsistent findings in previous research regarding the relationship between online authentic self-presentation and mental health. Previous research has yielded mixed findings regarding whether online authentic self-presentation benefits or undermines mental health. Some studies have emphasized its positive effects, showing that authentic self-expression on social media is associated with greater life satisfaction and subjective wellbeing, more positive affect, and fewer subsequent mental health symptoms (Bailey et al., 2020; Bunker et al., 2024; Reinecke and Trepte, 2014). The present study suggests that focusing solely on the direct path from online authentic self-presentation to depression may underestimate its actual influence. In fact, its effect appears to operate largely through the key mediating mechanism of relatedness need satisfaction. In other words, the inconsistencies in previous findings may be closely related to whether core psychological mechanisms such as relatedness need satisfaction were incorporated into the analytical model. At the same time, when considered in light of the real-life circumstances of Chinese university students, the buffering role of relatedness need satisfaction against depression may be particularly salient in a context characterized by intensified academic competition, employment pressure, and social comparison. This may also explain why the mediating effect observed in the present study demonstrated relatively strong explanatory power.

In addition, this study reflects, to some extent, the contextual characteristics of research on university students’ mental health in the digital age. At present, university students’ online social interaction is no longer confined to traditional text-based communication, but is increasingly embedded in media environments such as short videos, online communities, and livestream interactions, which are more immediate, visible, and participatory in nature. Against the backdrop of constantly evolving modes of online interaction, authentic self-presentation is more likely to elicit responses from others, generate emotional resonance, and foster group interaction, thereby improving the efficiency with which relatedness needs are satisfied (Martinez et al., 2025). For university students facing substantial academic competition and developmental pressure, the attainment of a sense of belonging may carry even greater psychological protective significance. Therefore, the present finding concerning the mediating role of relatedness need satisfaction not only deepens our understanding of the relationship between online authentic self-presentation and depression, but also provides new empirical support for interpreting university students’ psychological adaptation in digital contexts from the perspective of psychological need satisfaction.

### 5.3 The moderating effect of perceived value of team sports participation

The present study further examined the moderating role of perceived value of team sports participation in the mediating pathway linking online authentic self-presentation, relatedness need satisfaction, and depression. The results showed that, after controlling for demographic variables, the interaction term between online authentic self-presentation and perceived value of team sports participation significantly and negatively predicted relatedness need satisfaction (*B* = −0.12, *t* = −2.97, *p* < 0.01), and the interaction term between relatedness need satisfaction and perceived value of team sports participation also significantly and negatively predicted depression (*B* = −0.17, *t* = −3.45, *p* < 0.01), thereby supporting Hypotheses H3a and H3b.

With regard to the first half of the mediating pathway, the interaction between online authentic self-presentation and perceived value of team sports participation significantly and negatively predicted relatedness needs, indicating that perceived value of team sports participation weakened the positive effect of online authentic self-presentation on relatedness needs. In other words, compared with university students with lower levels of perceived value of team sports participation, those reporting higher levels showed a weaker dependence on online authentic self-presentation for relatedness need satisfaction. This result does not mean that online authentic self-presentation directly reduces relatedness need satisfaction. Rather, it suggests that the positive association between online authentic self-presentation and relatedness need satisfaction becomes weaker as the perceived value of team sports participation increases. Thus, perceived value of team sports participation may condition the sources from which university students experience relatedness need satisfaction, making it less strongly tied to online authentic self-presentation and more closely associated with offline interaction and group-based participation.

This finding is consistent with previous research showing that team sports participation contributes to the development of interpersonal competence, group identity, and team cohesion, thereby enhancing individuals’ sense of belonging (Wilson et al., 2018). However, prior studies have focused primarily on the direct beneficial effects of team sports participation on psychological health, while paying relatively limited attention to its contextual moderating function within the mechanisms through which online behavior influences mental health (Vega-Díaz and González-García, 2024). The findings of the present study help address this gap to some extent. They suggest that university students’ perceived value of team sports participation may be linked to meaningful offline social engagement and may function as an important real-life social context. In this context, the association between online self-expression and psychological need satisfaction may be shaped by students’ sport-related social perceptions.

Theoretically, this finding may be understood in light of the online psychological need satisfaction advantage perspective and social identity theory. Online authentic self-presentation may provide university students with opportunities to express their true selves and seek understanding and responses from others. However, such relatedness satisfaction often depends on mediated interaction contexts, and its stability and continuity may be relatively limited. In contrast, students who report higher perceived value of team sports participation may be more likely to recognize team sports as a meaningful offline cooperative context. Through shared training, cooperation, and sustained interaction, individuals may develop emotional bonds, trust, and group identity based on real-life experiences, thereby obtaining a deeper and more enduring sense of relatedness (Ni et al., 2025).

Regarding the second half of the indirect pathway, the interaction between relatedness need satisfaction and perceived value of team sports participation was significantly and negatively associated with depressive symptoms. This finding suggests that the negative association between relatedness need satisfaction and depressive symptoms became stronger as the perceived value of team sports participation increased. In other words, relatedness need satisfaction appeared to be more strongly linked to lower depressive symptoms among university students who reported higher perceived value of team sports participation, whereas this association was weaker among those with lower perceived value. Thus, perceived value of team sports participation may provide a social context in which relatedness need satisfaction is more closely associated with lower depressive symptoms.

From the perspectives of need to belong theory and social support theory, team sports may provide university students with concrete and real-life emotional support and social connectedness through immediate interaction, cooperative experiences, and relatively stable peer companionship during sport participation. Even when individuals’ relatedness needs are not fully satisfied, real-life support derived from team contexts may provide additional psychological resources and may be associated with lower levels of loneliness, alienation, and helplessness, which are important psychological correlates of depressive symptoms (Vashdi et al., 2024). In addition, successful cooperation, role involvement, and team identification in team sports may be associated with a stronger sense of self-worth and social efficacy, which may help individuals show better emotional regulation and psychological resilience when facing negative life events such as academic stress and interpersonal difficulties (Wei et al., 2025). Therefore, students with higher perceived value of team sports participation may be more likely to recognize or engage in sport-related social experiences, and relatedness need satisfaction may be more strongly linked to lower depressive symptoms. This interpretation is also broadly consistent with Cooney et al. (2013) conclusion that sport participation may be associated with lower depressive symptoms.

In summary, the present study found that perceived value of team sports participation played a significant moderating role at both stages of the pathway linking online authentic self-presentation, relatedness need satisfaction, and depression. This finding indicates that, as an important real-world protective factor, perceived value of team sports participation not only shapes the sources through which university students satisfy their relatedness needs, but also strengthens the buffering effect of relatedness need satisfaction on depression, thereby further refining the mechanism through which online authentic self-presentation influences depression.

### 5.4 Educational implications of perceived value of team sports participation for the effects of online authentic self-presentation on relatedness need satisfaction and depression among university students

**Figure 4:**
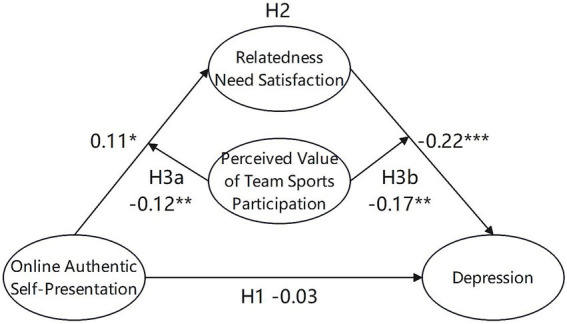
Moderated mediation model of perceived value of team sports participation in the association among online authentic self-presentation, relatedness need satisfaction, and depression. Values shown are path coefficients. *p < 0.05, **p < 0.01, ***p < 0.001.

Based on the findings of the present study (Figure 4), Chinese universities may advance mental health education and sport-related governance practices for university students in the following three respects. First, greater attention should be paid to changes in students’ behavioral patterns and psychological needs in the internet era. Rather than responding to their online social behavior solely through restrictive or didactic approaches, universities should adopt a developmental perspective to understand the prevalence of online authentic self-presentation and its potential psychological implications, while strengthening students’ sense of belonging in real life by enriching campus sports culture and creating more opportunities for offline social interaction. Second, universities should move beyond an educational orientation that overemphasizes individual competition and single-dimensional ability evaluation, and instead place greater value on students’ development through collective cooperation, role-taking, and group integration. In this regard, the positive role of team sports in enhancing team awareness, real-world belonging, and psychological adaptability should be fully recognized and utilized. Third, stronger coordination should be promoted among physical education, mental health education, and student management. Team sports should be incorporated into the university mental health promotion system, and regular participation mechanisms should be established through clubs, student organizations, and class-based activities, so as to further realize the comprehensive educational value of sport in strengthening the body, shaping personality, cultivating willpower, and promoting social adaptation.

## 6 Conclusion

1. Relatedness need satisfaction mediated the relationship between online authentic self-presentation and depression among university students; that is, online authentic self-presentation could indirectly reduce depression through relatedness need satisfaction.
2. Enhancing students’ perceived value of team sports participation may help shape the association between online authentic self-presentation and relatedness need satisfaction. At the same time, higher perceived value of team sports participation may strengthen the association between relatedness need satisfaction and depressive symptoms.
3. Universities should take into account the developmental characteristics of students’ physical and mental health in the digital age, strengthen the coordination among physical education, mental health education, and student governance, and fully leverage the comprehensive educational value of team sports in enhancing relatedness, promoting psychological adaptation, and fostering well-rounded personality development.

## 7 Limitations and prospects

Several limitations should be acknowledged. First, the cross-sectional design limits causal inference, and reciprocal or reverse relationships among the study variables cannot be excluded. Moreover, perceived value of team sports participation reflects students’ attitudes and evaluations rather than their actual behavioral involvement. Future studies should therefore adopt longitudinal or experimental designs and incorporate behavioral indicators such as participation frequency, duration, intensity, and type.

Second, participants were recruited mainly from key universities in first-tier cities, which may restrict the generalizability of the findings. Universities across different regions and city tiers may differ in educational resources, opportunities for team sports participation, student composition, and mental health support. Future research should include students from more diverse geographical areas and institutional backgrounds and further examine possible differences by gender, socioeconomic status, and urban–rural origin.

Third, although participants were drawn from six universities and preliminary comparisons showed no significant between-university differences, the nested structure of students within institutions was not explicitly modeled. Future studies involving a larger number of universities should employ multilevel models or cluster-robust methods to account for potential institutional effects.

Finally, several potentially important confounding variables were not comprehensively assessed, including general physical activity, individual sport participation, social media use intensity, offline social support, prior mental health status, socioeconomic background, and existing team membership. These factors may be associated with both the predictors and depressive symptoms and should therefore be included as covariates or potential moderators in future research.

Despite these limitations, the present study provides preliminary evidence on how online authentic self-presentation, relatedness need satisfaction, and the perceived value of team sports participation may jointly relate to depressive symptoms among university students. By linking online interpersonal experiences with the psychosocial value of offline team sports, the study offers a useful basis for future longitudinal, behavioral, and intervention-based research.

## Data Availability

The original contributions presented in the study are included in the article/supplementary material, further inquiries can be directed to the corresponding authors.
